# Mastering CT-based radiomic research in lung cancer: a practical guide from study design to critical appraisal

**DOI:** 10.1093/bjr/tqaf051

**Published:** 2025-03-18

**Authors:** Ashley Horne, Azadeh Abravan, Isabella Fornacon-Wood, James P B O’Connor, Gareth Price, Alan McWilliam, Corinne Faivre-Finn

**Affiliations:** Division of Cancer Sciences, The University of Manchester, Manchester, M13 9NT, United Kingdom; Department of Thoracic Oncology, The Christie NHS Foundation Trust, Manchester, M20 4BX, United Kingdom; Division of Cancer Sciences, The University of Manchester, Manchester, M13 9NT, United Kingdom; Division of Cancer Sciences, The University of Manchester, Manchester, M13 9NT, United Kingdom; Division of Cancer Sciences, The University of Manchester, Manchester, M13 9NT, United Kingdom; Division of Radiotherapy and Imaging, Institute of Cancer Research, London, SW7 3RP, United Kingdom; Division of Cancer Sciences, The University of Manchester, Manchester, M13 9NT, United Kingdom; Division of Cancer Sciences, The University of Manchester, Manchester, M13 9NT, United Kingdom; Division of Cancer Sciences, The University of Manchester, Manchester, M13 9NT, United Kingdom; Department of Thoracic Oncology, The Christie NHS Foundation Trust, Manchester, M20 4BX, United Kingdom

**Keywords:** lung cancer, radiomic, early-career researchers, guide

## Abstract

Radiomics is a health technology that has the potential to extract clinically meaningful biomarkers from standard of care imaging. Despite a wealth of exploratory analysis performed on scans acquired from patients with lung cancer and existing guidelines describing some of the key steps, no radiomic-based biomarker has been widely accepted. This is primarily due to limitations with methodology, data analysis, and interpretation of the available studies. There is currently a lack of guidance relating to the entire radiomic workflow from study design to critical appraisal. This guide, written with early career lung cancer researchers, describes a more complete radiomic workflow. Lung cancer image analysis is the focus due to some of the unique challenges encountered such as patient movement from breathing. The guide will focus on CT imaging as these are the most common scans performed on patients with lung cancer. The aim of this article is to support the production of high-quality research that has the potential to positively impact outcome of patients with lung cancer.

## Introduction

Lung cancer is the leading cause of cancer-related mortality worldwide.^1^ Despite diagnostic and treatment innovations, outcomes remain disappointing, and many patients experience treatment failure following curative-intent treatments.^2^ Treatments includes surgery, radiotherapy, and drug treatments and are offered based on cancer stage and patient fitness.^3^ Patients with stage I-III disease with good performance status can be offered curative-intent surgery- or radiotherapy-based treatments. Local and distant control rates improve with multi-modality approaches, though they are associated with higher toxicity rates.^4^ As a result, the goal of delivering personalized precision healthcare remains unfulfilled.^5^

CT scans are performed routinely in patients with lung cancer, with emphasis on identifying and characterizing primary lung, nodal, and metastatic lesions and quantifying changes in dimension and volume. Since images are 3D greyscale data, this can be mined, a process known as radiomics, to extract a variety of image-based features that describe the spread and spatial distribution of data in each voxel.^6^ These features can then be assessed as a prognostic or predictive biomarker. However, due to varying research quality, a lack of reproducibility, non-standardized methods, and limited wider clinician awareness and acceptance, it remains a tool that is used within research. This practical guide aims to address an important gap in the literature by providing support to early career researchers in designing and conducting high-quality radiomic research. Lung cancer CT imaging is the focus of this guide because they are the most frequently performed scan on patients with lung cancer and as a result there is a large amount of mineable data. In addition, there are unique challenges to consider, such as movement caused from patient breathing.

Established baseline patient-, tumour-, and treatment-related features are known to be associated with clinical outcomes following curative-intent treatment for lung cancer (see Supplementary Table 1 for summary).^7^^,^^8^ However, these features lack sensitivity, and there is a critical need to develop biological markers that will allow patients to be offered personalized treatments.^5^ This includes informing discussions around expectations of treatment and the risk of significant toxicity. There is also a need to identify patients who will benefit from consolidation drug therapies following curative-intent treatment. Despite a wealth of published research, there is currently no widely accepted biomarker to guide the curative-intent management of patients with lung cancer.^5^

### Imaging-based biomarkers and radiomic features

CT thorax/abdomen scans are the most frequently performed imaging modality during a lung cancer disease course. Scans are analysed by radiologists who report on a priori qualitative features and simple quantitative measurements. A significant amount of complex quantitative imaging data is not measured, and their significance is not well established. There is an opportunity to increase the utility of routinely acquired CT scans by identifying imaging-based biomarkers.

The current explosion in radiomics studies dates to 2012, where Lambin et al^9^ described how feature analysis could be deployed in a multi-step workflow or pipeline.^9^ Delta-radiomics describes the analysis of two or more scans from different timepoints and allows inter-scan feature variation to be assessed. Different modality scans can also be analysed together. Two main approaches to image analysis and feature extraction have been developed:

- Traditional methods extract potentially hundreds to thousands of pre-determined or handcrafted features from a scan.^10^ Features are split into three categories: morphological/shape, first-order/intensity, and second-order/textural (summarized in Table 1). Extracted features are grouped together to assess for association with a chosen clinical feature or outcome.
- Featureless methods integrates deep learning (DL) algorithms to extract “discovered” or learnt features relevant to a predefined clinical feature or outcome.^11^ DL techniques, such as convolutional neural networks, are commonly used and compared to traditional methods do not require explicit handcrafted feature engineering.^12^

**Table 1.** Summary of traditional handcrafted radiomic feature categories.

Radiomic feature categories	Definition	Graphical representation
Morphological/shape features	Describes geometric features that are either based on length, area, or volume. They should be calculated using 3D analysis rather than on single planes. The features can be calculated based on the number of voxels (voxel counting), coordinates, or distance from the ROI mask surface, known as the ROI mesh.	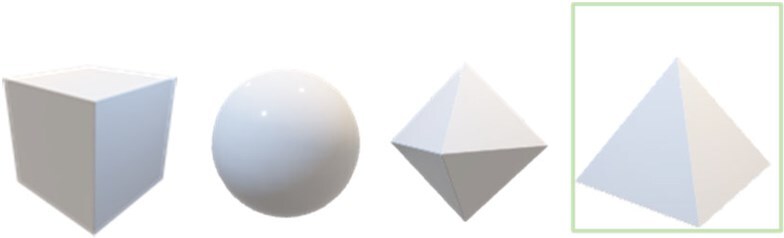
First-order/intensity features	Describes features based on voxel intensity or brightness. These measures require discretization steps to effectively summarize. Intensity is recorded differently depending on modality. For example, CT scan distributions are discrete, while PET-CT are continuous, and MRI uses arbitrary units. As a result, different methods are required.	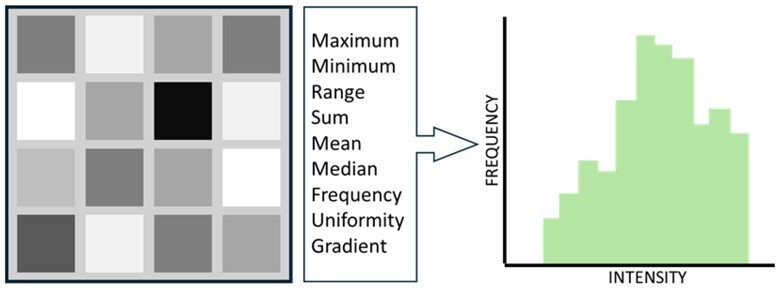
Second- or higher-order/textural features	Describes complex features that assesses texture. This includes the distribution of discretized grey level or variation of intensity between voxels. The data are aggregated together to summarize intensity data across a length, area, or volume.	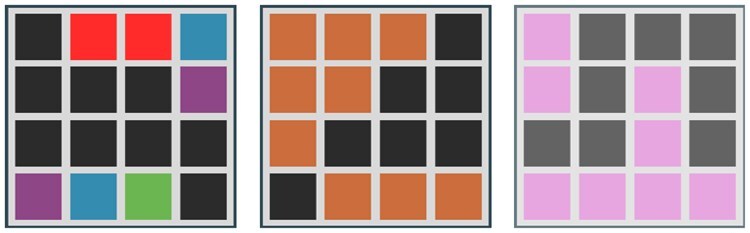

Abbreviation: ROI = region of interest.

Radiomics comes with several distinct advantages compared with other novel biomarker technologies. It is highly automated, non-invasive, and makes use of routine imaging.^5^^,^^9^ Additionally, as the whole tumour is analysed, rather than a portion acquired from a biopsy, the features extracted could measure intra-tumour heterogeneity.^13^ For these reasons, it has the potential to be cost-effective, acceptable to patients, and highly informative.

Despite an exponential increase in the number of published research papers, there is currently a lack of confidence in integrating radiomic features into clinical practice. This lack of progress is multifactorial but includes issues related to inconsistent methods producing unreproducible non-validated results. Some key aspects of technical validation, such as measuring test-retest precision, are lacking in most tumour types, although some studies are beginning to address this gap.^14^^,^^15^ While the notion that intra-tumoral heterogeneity relates to outcome has face validity, the rationale linking specific abstract imaging features to biological process is harder to understand. There is also limited research integrating clinical and biological data. This makes it difficult to compare how radiomic features perform compared to known predictors, makes interpretation challenging, and makes it impossible to assess potential clinical applications. Several radiomic review articles were identified that have appraised the quality of curative-intent lung cancer studies.^6^^,^^16–19^ Most of the included studies were radiotherapy-based, and no review was identified that focused on surgical studies. Recurrent limitations identified are summarized in Table 2.

**Table 2.** Summary of recurrent research and methodological limitations identified from lung cancer radiomic studies.

Recurrent research and methodological limitations:
Patient cohort: Heterogeneous cancer stage Heterogeneous pathology Small cohort sizes Retrospective cohorts
Imaging-related: Different scanners, acquisition, and reconstruction protocols Consideration of patient movement during image acquisition Consideration of impact of segmentation method used
Radiotherapy-based treatments: Heterogeneous dose fractionation Different radiotherapy techniques Use of systemic therapies
Radiomic analysis: Limited methodological transparency Limited justification of method or software use Limited description of plan for missing data Extraction of large number of features Inclusion of non-stable radiomic features Lack of external validation Limited discrimination and calibration statistics
Miscellaneous: Limited biological rationale of results Lack of cost calculations Limited focus on toxicity No use of patient-reported outcome measures Limited open science and data

In an attempt to standardize the radiomic workflow, internationally agreed standards and a “how to guide” have been published.^20–22^ The radiomic quality score (RQS) version 1.0 is a 16-point framework that assesses methodology, analysis, and statistical reporting (summarized in Supplementary Table 2).^20^^,^^23^ A second version is under development, which will contain a more extensive 36-point framework and will allow for DL- and handcrafted-based features to be assessed. The Image Biomarker Standardisation Initiative (IBSI) is an international collaboration that aims to standardize key nomenclature and feature definitions.^21^ It includes the benchmarking of feature extraction and image processing methods and has been adopted by leading research institutions. The “how to guide” is tumour agnostic and offers additional practical support for core aspects of the workflow.^22^

Guidelines and reviews should be used to support the design, structure, and appraisal of radiomic research. However, key parts of the workflow are not well described. For example, they offer limited focus on study design, cohort selection, image acquisition, model building, validation processes, and clinical application.

### The promising value of radiomics in lung cancer

**Figure 1.**
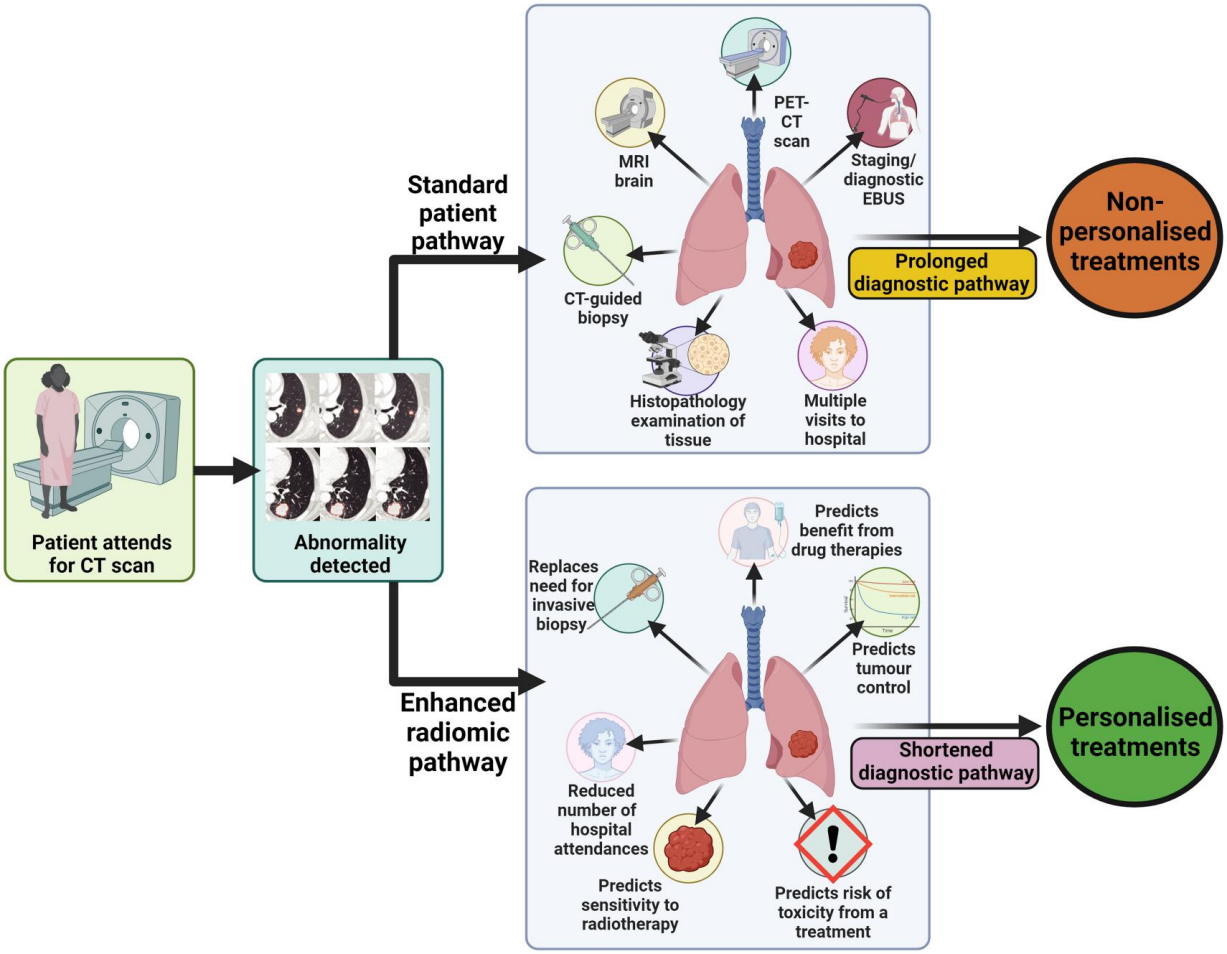
Potential advantages of integrating radiomic image analysis in lung cancer diagnostic and management pathways^a^. ^a^Created with BioRender.com.

There is an opportunity for radiomic-based imaging features to find a role in supporting personalized diagnostic and management decisions in patients with lung cancer (key potential advantages summarized in Figure 1).^5^^,^^23^ This could include reducing the reliance on biopsies by identifying relevant pathological information such as tumour subtype and PD-L1 status.^24^^,^^25^ This would benefit patients in situations where biopsy is not pursued due to frailty or tumour location. Additionally, radiomics could be used to more accurately stage patients, predict occult lymph node metastasis, and enhance or replace the need for certain staging investigations.^26–28^ This in turn could shorten the time to definitive management and reduce hospital visits. Radiomics features could also support clinical decision-making by predicting tumour control rates, risk of significant toxicity, quantifying the benefit of the addition of drug therapies, and informing discussions around treatment futility.^6^^,^^16–19^^,^^29^

### A more complete radiomic workflow: study design to critical appraisal

**Figure 2.**
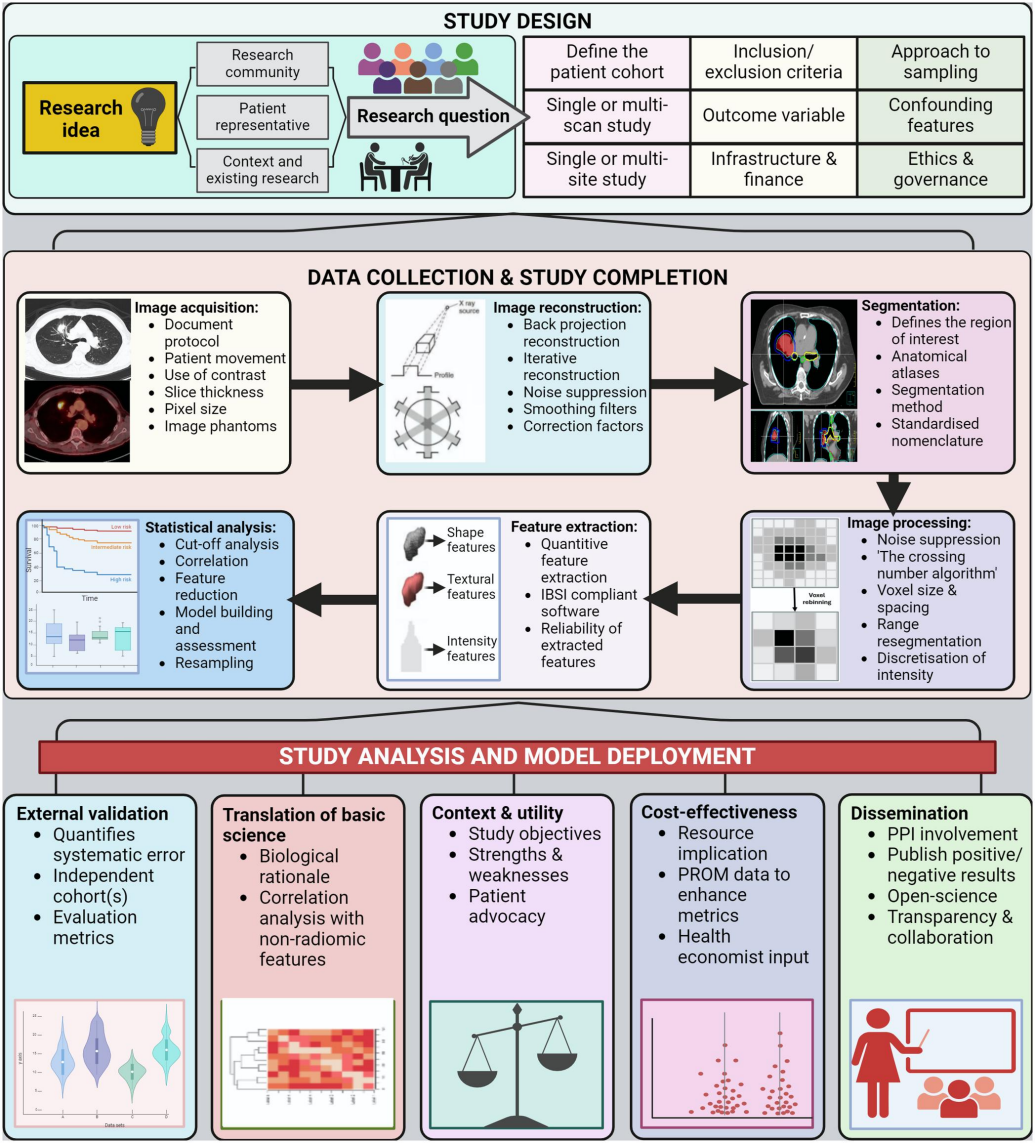
Proposed radiomic workflow^a^. ^a^Created with BioRender.com. Abbreviations: IBSI = Image Biomarker Standardisation Initiative; PROM = patient-reported outcome measure.

The authorship team, which includes early career researchers, identified a gap in the literature to support inexperienced researchers in performing their own independent radiomic research. In this paper, we aim to address this gap by proposing a comprehensive and practical radiomic workflow. We have aligned it with existing guidelines but have taken a more complete approach by starting with study design and ending with critical appraisal (see Figure 2). Relevant concepts from the RQS, IBSI, and manuscripts demonstrating methodology are highlighted to demonstrate synergy, good practice, and for reference. Additionally, we have chosen to focus on a single imaging modality and anatomical region, acknowledging the distinct approaches and considerations involved when interpreting and analysing lung cancer CT scans. Key issues are as follows:

- *Patient characteristics:* many patients with lung cancer present with additional imaging findings, for example, atelectasis, emphysema, fibrosis, and changes from prior radiotherapy or surgery.
- *Histological variability:* lung cancer encompasses different subtypes, each with distinct biological behaviour, imaging features, and response to treatments.
- *Tumour extent and position:* lung cancers can be surrounded by normal lung or involve adjacent structures such as blood vessels and the chest wall. Additionally, local lung collapse can obscure tumour boundaries, making differentiation from surrounding consolidation challenging.
- *Imaging variability:* differences in acquisition parameters, such as slice thickness, use of contrast and reconstruction algorithms, can introduce variations in imaging protocols.
- *Tumour motion:* respiration and patient movement can lead to inconsistences in imaging data, impacting tumour visualization, segmentation, and measurement accuracy.
- *Dependency on segmentation:* variability in tumour segmentation methods, particularly for complex or poorly defined tumours, affects measurement accuracy and reproducibility, leading to potential inconsistencies in radiomic feature extraction.

Ultimately, the intention is to support the production of high-quality standardized research that contributes meaningfully to the evidence base. To demonstrate the applicability of the workflow, it has been applied to two cohorts of patients undergoing curative-intent radiotherapy for non-small-cell lung cancer (NSCLC) in our institution. This includes a retrospective real-world cohort and a cohort from a prospective multiomic biomarker study.^30^

## Study design

Discussing study design in detail is beyond the scope of this article. However, we believe a pragmatic and considered approach should be adopted to produce robust research output. As a result, it is a process that early career researchers should actively engage in. There are a number of study design guides, including one that is specific to lung cancer that was published in the *Journal of Thoracic Oncology*, that can be referred to.^31^^,^^32^ To compliment these guides, we have reflected on our own and other radiomic research and have constructed a list of what we consider, mandatory considerations (see Table 3). This includes key requirements such as engaging with patient advocates and relevant experts and setting an appropriate and valid study question. Defining the study cohort and patient sampling approaches will be influenced by the research question, the study aims, data availability, funding, and the study timeline.

**Table 3.** Mandatory considerations of study design in radiomic research.

Mandatory considerations of study design:
Engaging with members of the research community: Relevant leading and local experts Early career researchers who share a similar research focus Attending relevant national and international conferences Promotes information sharing Supports the formation of collaborative networks
2. Public patient involvement: Enhances different aspects of study design Improves research relevancy Increases the likelihood that the study will be acceptable to patients and patient-centred Should support writing protocol and patient facing documents Invaluable when applying for funding Consider novel methods to engagement, for example, using a citizens jury approach^33^
3. Research question setting requires: Adopting a pragmatic approach Basing any question in the current clinical context and existing research base Identifying current knowledge gaps and why they need addressing Highlighting synergy between existing research with the proposed research Engaging with clinical colleagues to specify clinically useful research questions Increases the likelihood that research is value for money
4. Defining the study cohort requires: Identifying relevant inclusion/exclusion criteria Single or multi-scan study: mixed modality or delta-radiomic approach Single or multi-site study Opportunity to ensure study cohort is representative of a real-world population
5. Patient sampling approaches (**RQS 11**): Retrospective: most common, good demonstration of methodology, but rarely reproducible and therefore should be interpreted with caution Prospective: reduced risk of bias, describe temporal sequences and causality (if an interventional study is being performed), but time and financial costs are high and patients can be lost to follow-up Repurposing of an existing prospective dataset: likely to be comprehensive and accurate and avoids the time, resource, and financial cost of performing a de novo study. If dataset is from a completed study, the eligibility criteria could be restrictive and not be representative of a real-world population. The dataset will also include patients receiving experimental treatments that may never be approved Institutional registry databases: institution-based secure data or trusted research environments, such as the UK Computer Aided Theragnostics project, exist that allow researchers to access large amounts of anonymized patient data from electronic patient records for real-world data studies.^34^ Having umbrella ethics allows research to be performed efficiently Combination of approaches: a considered approach combining different datasets while acknowledging the strengths and weaknesses of each cohort might be most efficient and appropriate
6. Outcomes and plan for data collection requires (**RQS 6**): A rational approach Identifying a clinically useful outcome variable Collecting confounding features: known predictors or prognostic features Defining time variables clearly from outset, for example, date from histological diagnosis Considering inclusion of patient-reported outcomes measures Considering inclusion of cost-related measures Justifying any additional burden to patients If a multi-site approach has been taken, consider a centralized or federated approach to data collection
7. Statistician support can be used: To perform power calculations and design the data collection plan To design a prospective statistical analysis plan To perform an independent statistical review
8. Ethics and governance: Important to ensure all regulatory approvals are in place prior to commencement Ensure there is a plan for data anonymization Explicitly state any plan or anticipation of data sharing Registration of any prospective or retrospective study on a trials database, such as the National Library of Medicine (NLH) Clinical Trials database or the EU Clinical Trials Register

Abbreviation: RQS = radiomic quality score.

## Data collection and study analysis

Once the above aspects have been considered and governance procedures have been completed, it is then possible to proceed with data collection and study analysis. The steps (shown diagrammatically in Figure 2) include the following:

### Image acquisition, feature variability, and test-retest experiments (RQS 1,3-4)

Image acquisition is the first step in the workflow described by the RQS and IBSI. It describes the process where a scan is undertaken, and digital imaging data are electronically stored. Sources of variation are: (1) acquisition protocols, (2) scanning hardware, (3) the use of contrast, particularly for non-shape based features,^35^ and (4) patient movement, such as from breathing. Thorax scans are commonly performed with the patient free breathing. Images are acquired at different points of the breathing cycle (4D) or are combined to create composite images, such as the maximum intensity projection. Key documentation includes imaging modality, institution, type of scanner, and the type of image in relation to the breathing cycle. Specific to CT scans slice thickness, pixel size, bit greyscale resolution, peak tube voltage, and tube current also needs to be documented.

Variability over short time intervals is assessed with test-retest experiments. There is limited research assessing changes in features between diagnostic CT and cone beam CT (CBCT) scans acquired from patients with lung cancer, however this has been assessed in other cancer types.^14^^,^^36^ Other test-retest experiments have been performed assessing stability between images acquired at different points of the breathing cycle of thorax 4D-CT scans.^15^^,^^37^

Methods to reduce image acquisition feature variability includes:

- Image phantoms can be used across all scanners to analyse inter-scan variation and calculate correction factors.^38^ Practically, this step becomes difficult to perform if images are being acquired from multiple scanners across different institutes and if retrospective analysis is being performed. Image phantoms are therefore more easily integrated into smaller exploratory prospective studies with a finite number of scanners.
- An alternative is to only include imaging features that are stable to changes in imaging protocol and scanner. Feature stability between CT scanners is described elsewhere.^39^
- Test-retest experiments can be used to assess temporal stability, for example, of a “radiomics signature.”
- DL-based correction algorithms can also be used to standardize the imaging data prior to reconstruction. Different algorithms have been developed and other research groups methods can be reviewed.^40^ Image synthesis algorithms can improve the image quality of CBCT scans.^41^ Whether this generates images suitable for radiomic analysis remains to be seen and radiomic features extracted from these scans will need to be compared with those extracted from diagnostic scans.

Methods to reduce IV contrast related feature variability include:

- Choosing to exclude scans where IV contrast was/was not administered for analysis. Previous research may influence this decision.
- Alternatively, the analysis can be focused on radiomic features that demonstrate stability regardless of whether IV contrast was administered. However, there does not appear to be any significant research investigating this.

### Image reconstruction (RQS 1,3)

Image reconstruction describes the process that converts data collected during image acquisition into 2D and 3D images. This is usually performed using filtered back projection reconstruction algorithms although this method is associated with streak and noise artifacts.^42^ As an alternative, iterative reconstruction algorithms are now available that use noise suppression approaches to produce higher quality images.^43^ Choice of reconstruction kernels to sharpen specific anatomical structures also affects the final image.^44^ Reconstruction protocol and choice of kernel introduce a further source of image variation.

Methods to reduce reconstruction variability:

- Access source image acquisition data, rather than reconstructed images, and perform image reconstruction using a standardized protocol to ensure equal slice thickness, pixel/voxel dimensions, and reconstruction methods.
- Where image acquisition data are not available, then other methods are required. These include smoothing filters, the use of correction factors, and DL approaches (see Image Processing section).
- Excluding images from scans where reconstruction parameters are outside of a preset criterion.

### Segmentation of regions of interest (RQS 2)

While an entire image can be analysed, more commonly a region of interest (ROI) needs to be identified for analysis. The contents of an ROI depend on the aim of the analysis. For example, an ROI could be the tumour for genomic or clinical outcomes studies or organs at risk for toxicity prediction studies. Manual, semi-automated, and automated segmentation describe the three main approaches to identify an ROI:

- *Manual segmentation methods:* software is used to manually outline anatomy on each 2D slice to create a 3D volume. It is an integral step in the radiotherapy planning process and can be used to calculate volumes as part of the image reporting process. In a clinical setting, it requires a trained specialist, is time intensive, and demonstrates high intra- and inter-operator variability.^45^ Patients who are planned for radiotherapy will usually have manual segmentation of relevant structures, and there is an opportunity to use these for analysis. Peer review and quality assurance processes varies, so it is important to assess whether these processes have been conducted and understand their specific procedures.
- *Semi-automated segmentation:* describes different methods that combine automated tools with clinician input. They are commonly used to make radiotherapy planning more efficient and accurate. Tools include interpolation that complete a volume by adding contours to intentional gaps left between manually volumed 2D slices.^46^ Other tools generate an expanded (or reduced) volume based on an existing ROI.^47^ The size increase (or decrease) can be uniform in all directions or can be set differently in each planar direction. More complex algorithm-based tools are also available that can generate a whole volume, such as for the lungs.^48^ Usually, clinical input is required initially to identify a geographical landmark and then for editing or feedback once the volume has been produced.
- *Automated segmentation methods:* algorithm-based tools can independently and automatically define the ROI without supervision.^47^^,^^49^ As a result, they are operationally time efficient. A recent survey demonstrated that the safe integration of automated tools into radiotherapy planning was supported by the majority of radiation oncologists.^50^ However, in their current form, no fully automated segmentation method is validated for clinical use and should only be used within academic studies.

### Defining ROIs and standardized nomenclature

ROIs require consistent nomenclature and definitions as to their contents. This can be a particular issue for retrospective radiotherapy-based studies. This is because variation will have been introduced when different non-standardized protocols are followed. For example, visible tumour can be labelled as macroscopic tumour volume or gross tumour volume. Variation is also introduced when ROI contents is not standardized. For example, in stage III NSCLC, the analysed volume is frequently limited to the primary tumour, although some studies include regional lymph node metastasis. This is an important consideration as certain radiomic features, such as sphericity, are sensitive to whether nodal disease is included or not.^51^ However, the decision-making behind this is frequently not documented and so it is not clear whether this was a decision based on practical or biological reasoning.

A practical reason to adjust the ROI is when CBCT scans are included. In this situation, it is generally agreed that due to inferior image quality, lymph node metastasis should be excluded. A biological reason to adjust an ROI includes adding a margin around the macroscopic tumour.^52^ It is hypothesized that this expanded volume represents the tumour micro-environment. Features extracted from this region could describe immune and vascular mechanisms that influence response to radiotherapy, immune cell infiltration, and drug delivery.

Methods to reduce or manage ROI segmentation variability partly depend on the segmentation method used:

- Segmentation should follow established guidelines and atlases that defines anatomical landmarks and boundaries, such as those produced by the SABR UK Consortium.^53^
- Standardized nomenclature and definitions should be used as defined by a single protocol.
- Incorporating validated automated or semi-automated processes that will produce standardized reproducible segmentation.
- If a segmented volume is being adjusted to form a new ROI, then the protocol needs to state whether editing is required. For example, if a tumour volume is expanded and the new ROI includes structures such as the chest wall, then these structures might need editing out of the ROI if only lung parenchyma is to be analysed or included if tumour invasion forms part of the analysis.
- Quality assurance processes should be documented. Manual segmentation that undergoes a local or external peer-review process is likely to be of a higher standard than those that do not. In some radiotherapy clinical trials, this process might be mandated by the study protocol.
- If peer review is not available or not consistently documented, then other quality assurance measures should be considered. For example, a random sample of scans can be selected, and a second set of ROIs produced through a different method. This could include manual segmentation from an independent practitioner, or an alternative semi-automated or automated method. The similarity of the segmentation can then be calculated using the intraclass correlation coefficient, which measures the variation in extracted features, and overlapping rates.^54^
- When a multi-scan analysis is being performed, then consideration of how the ROI will be defined on each scan is required. Each scan can be considered separately, and a segmentation process would need to be performed on each scan. Performing manual segmentation (and quality assurance) for every scan is potentially time intensive and so semi-automatic and automatic approaches previously described should be considered. Alternatively, segmentation from one scan to another can be transferred using a soft tissue or bony landmark match. This is time efficient, but there will be variation in position and size of ROI that would not be accounted for.

### Image processing (RQS 1, IBSI guidance)

Image processing is a multi-step process that aims to improve image quality through data conversion, noise reduction, and harmonization steps.^9^ The IBSI describes a methodology, and this should be considered the current standard—key steps are summarized in Table 4.^21^ Different image processing software is available to perform this process. The steps required vary depending on imaging modality and image acquisition process.

**Table 4.** Summary of key steps in image processing as per the image biomarker standardization initiative (IBSI) guidelines.^21^

Image processing steps	Description
De-noising processing/noise suppression or reduction	Describes different filtering algorithms that reduce noise found within reconstructed images. The algorithm used depends on modality, the source of noise and include both spatial and frequency filters. Random noise is uniform across an image and is assessed during test-retest experiments. Random noise has less of an impact on analysis when compared to noise caused by metal implants, such as the image distortion produced from a cardiac pacemaker.
“The crossing number algorithm”	Algorithms are used to decide whether voxels on the edge of an ROI should be included or excluded for analysis. They consider several factors, such as origin, positioning and direction.
Standardization of voxel sizes and spacing	Analysis is sensitive to voxel size and so standardization is required. This is common when scans from multiple institutes are being analysed as local reconstruction protocols can differ. Different interpolation algorithms are available to transform the size of the voxels within an ROI. Methods include: Down-sampling increases the size of the voxels. As information across multiple voxels is merged, information is lost. Up-sampling decreases the size of voxel. As multiple smaller voxels are generated from larger voxels, artificial information is introduced through inference and image synthesis. Multiple-scaling combines up- and down-sampling methods to overcome the limitations from only using one.
Range re-segmentation	Describes a process that removes artifacts such as air and bone from images. Artifacts are filtered out by removing voxels that fall outside a pre-specified range of greyscale. This removes artifacts such as air and bone from CT and PET-CT scans. The equivalent step performed on MRI scans, where data are given arbitrary intensity units, is known as outlier filtering.
Discretization/normalization or voxel depth re-binning of image intensities	Bin values are averaged to smooth the data distribution. It enables tractable feature calculation, preserves differences across a feature distribution, and is another method to suppress noise. The histogram distribution can be adjusted by changing the number of bins or the width of bins. If too wide or too few bins are set, then features can be averaged out and lost. If too narrow or too many bins are set, then features can become indistinguishable from surrounding noise.

Abbreviation: ROI = region of interest.

Methods to reduce image processing variability:

- Following a systematic approach such those described by the IBSI guidelines.
- Documenting processing steps, any adjustment made, and the reasoning behind it.
- Choosing to use community accepted open-source software such as the 3D-slicer plugin for PyRadiomics ensuring the name and version is recorded.
- Documenting software name and version.

### Feature extraction or calculation (IBSI guidance)

Describes the conversion of imaging data into quantifiable features. There are two methodological approaches—traditional and featureless. The IBSI provides guidance on how to perform traditional handcrafted feature extraction, including additional image processing methods for extracting handcrafted features.^21^ Features are grouped together by type, such as morphological-, intensity-, or textual-based (summarized in Table 2 with a more detailed summary in Supplementary Table 3). ROI mask features are usually based on morphology and intensity, as well as position and relative grid distances, feature aggregation, and distance weighting methods.

Different extraction platforms are available, and while some are IBSI-compliant, there is no consensus on the optimum choice. These platforms include in-house software developed by individual institutions and therefore not always freely accessible, as well as open-source and commercial platforms. There is limited research comparing software performance and reliability. Most comparison studies use retrospective cohorts and focus on scans of patients with head and neck cancer.^55^^,^^56^ Only three studies with an exclusively lung cancer population were identified.^57–59^ One study used three different software platforms to build models to predict EGFR status.^58^ Different features were selected, suggesting reliability between software was poor. The other studies are currently in progress. The first includes a prospective cohort and aims to build different radiomic models to predict clinical outcomes.^57^ The second is a retrospective analysis that is using different methods to extract features from pre-operative PET-CT scans.^59^ These features are being analysed alongside gene mutations and circulating tumour cells to build a model to predict clinical outcome. The reliability of feature extraction software is further complicated by the identification of coding mistakes and the release of new versions with updated code.^55^

Featureless extraction describes an alternative approach that uses DL models, such as convolutional neural networks, to analyse an imaging dataset.^11^^,^^12^^,^^60^ Unlike traditional radiomics, which extracts predefined handcrafted features, convolutional neural networks are used to learn and extract features directly from the 3D volumetric imaging data. Each network consists of artificial neurons that recognize different shapes, texture, and structural patterns. The networks can be trained using:

- *Supervised learning:* the model is trained using labelled imaging data paired with the known outcome of interest.^11^
- *Unsupervised learning:* the model learns patterns without predefined labels, discovering latent structures within the dataset.^11^
- *Semi-supervised learning:* a hybrid approach where a small portion of labelled data is used to guide the learning process while leveraging a larger set of unlabelled data.^11^ This is particularly useful in medical imaging, where obtaining high-quality labelled datasets is resource-intensive and time-consuming.

Additionally, DL methods can minimize human involvement by analysing the entire imaging dataset without the need for manual ROI segmentation.^12^^,^^60^ This can reduce segmentation variability but also introduces challenges, such as ensuring robust dataset annotation, computational efficiency, and interpretability. In general, DL-based methods require large, well-annotated datasets, computational resources, and specialized expertise. Features are learned progressively through multiple layers of a neural network and can be divided into three levels of increasing complexity:

1. *Low-level features:* basic patterns that are detected in early network layers and represent edges, textures and intensity gradients.^60^
2. *Mid-level features:* formed by integrating low-level features into more complex representations.^60^ These mid-level features encode structural information, such as shape structures, localized texture, and spatial intensity patterns.
3. *High-level features:* complex, non-linear representations learnt in fully connected layers of a neural network, integrating information across the entire ROI.^60^ These high-dimensional features are often abstract, making interpretation challenging. To aid interpretability, results can be displayed graphically using visualization techniques such as saliency maps (eg, Grad-CAM, LIME), which highlight ROIs within the imaging data and provide insights into the model’s decision-making process.^61^

Methods to reduce feature extraction variation:

- Using an open-source IBSI-compliant software platform, ensuring the name and version is recorded. PyRadiomics and MATLAB are the most commonly used to extract features from CT scans performed on patients with lung cancer treated with radiotherapy.^17^^,^^62^
- Consider assessing reliability of extracted features using multiple IBSI-compliant software platforms.
- Ensure feature calculation hyper-parameters are reported and, if possible, harmonized to common default values used across different packages.
- Using guidelines, such as those published by IBSI, to ensure a standardized nomenclature and feature extraction method.
- Avoiding features that have shown low reproducibility, such as those identified by IBSI.
- The RQS 2.0 (under development) will include further guidance for handcrafted and DL feature extraction.^20^ When published, it will promote a “FUTURE-AI” (Fairness, Universality, Traceability, Usability, Robustness and Explainability) approach, which should provide further guidance as to how to reduce feature extraction variation.

### Statistical analysis and assigning significance (RQS 4-10, IBSI guidance)

Statistical analysis is performed to identify associations between the extracted features and outcomes. The TRIPOD statement is a 22-point checklist that can be used to support the development of prediction models in healthcare.^63^ Some of these steps are highlighted by the RQS and the IBSI. Key steps are summarized in Table 5.

**Table 5.** Key steps in the statistical analysis of radiomic features.

Key steps	Description
Data collection and engineering:	
Patient and tumour clinical data collection	Including clinical biological and molecular features improves robustness and model performance and their inclusion should be considered necessary.^64^ Other novel health technology data can also be integrated. This includes patient-reported outcome measures (PROMs) and circulating-tumour DNA analysis such as within the VIGILANCE study.^30^ The specific data depend on the chosen model outcome and should include known predictors/confounding features—see Table 1. It is important to assess whether radiomic features are additive and exhibit stronger associations with the outcome when compared to known predictors. The completion and reliability of the data is an important consideration. Methods to compensate for missing or unknown data depend on whether the data were collected prospectively or retrospectively, the data type, and choice of modelling performed.^65^
Assessing variables:	
Cut-off analysis and reference values	Cut-off analysis determines the threshold value (cut-off point) that best separates different risk categories within a dataset. There are three main approaches to ascribe significance to feature values. A combination of these methods might be required to fully understand the significance of each feature and include: IBSI reference ranges: The IBSI provides reference ranges for features that can inform cut-off analysis. It also scores how well validated each reference range is. This allows an analysis to be focused on features that have previously demonstrated robustness. Median values: Cut-off analysis can be performed using the median value to separate patients into risk groups. Continuous variables: Time-to-event analysis and receiver operating characteristic (ROC) curves are both used to assess the risk distribution of a variable.^66^ Time-to-event analysis is used to examine the relationship between a variable and the time until the event occurs (eg, treatment failure). ROC curves are used to evaluate the diagnostic performance of a binary classifier (eg, treatment failure vs treatment success). Risk can be considered a dynamic process, although cut-offs can be introduced allowing a sample to be divided into risk groups if required.
Feature correlation/association	Describes the process of assessing for and measuring the magnitude of correlation or association between different radiomic features and/or with a clinical outcome. Traditionally, univariate and multivariate cox regression approaches have been used; however, ML methods are increasingly being adopted.^67^ To allow for a more comprehensive analysis, clinical variables and known predictors should be included.
Feature selection	Feature selection/reduction or dimension reduction describes methods that identify the most relevant and informative features without losing statistically significant information. Traditional and ML methods are available (see below). Single or multiple selection methods can be used. Features are divided into one of three categories: relevant, irrelevant, or redundant. Where relevant are statistically significant stable features, irrelevant are non-informative, and redundant are those that provide information that is already captured by other features and do not give additional statistical information.
Traditional feature selection	Different traditional statistical methods can be used to evaluate features based on their intrinsic properties. Common techniques includes clustering, intraclass, and concordance correlation coefficients and least absolute shrinkage and selection operator (LASSO) regression.^62^^,^^68^
ML feature selection	ML features selection methods are computationally more complex when compared to traditional methods and can be divided into filter, wrapper, or embedded approaches: Filter methods: describes processes where features are scored based on relevance to the outcome of interest. Features can then be ranked based on their scores and a threshold applied to select the best performing features.^69^ Wrapper methods: use an iterative search process in which different subsets of features are evaluated to identify the most predictive combination.^69^ This approach accounts for interactions between features but can be computationally intensive, especially in high-dimensional datasets, where the number of possible feature combinations grows exponentially. This makes them more prone to overfitting in small datasets compared to embedded methods. Common techniques include: Forward selection: start with no features selected and iteratively adds the best performing features. Backward elimination: start with all features included and progressively removes the least predictive ones. Exhaustive search: evaluates all possible subsets of features, making it the most computationally expensive method. Embedded methods: use a supervised learning process where features selection and model training are combined.^69^ As a result, they can be computationally efficient and are less prone to producing overfitted models compared to wrapper methods. Common techniques include: L1 regularization (LASSO regression): applies penalties that force the coefficients of less relevant features to zero, effectively eliminating them. This results in a sparse model built with only the most important features. L2 regularization (ridge regression): applies penalties to the squared values of coefficients, shrinking them without setting them to zero. This ensures all features are retained, with weighting indicating their relative importance. Elastic net: combines L1 and L2 regularization to balance feature selection (L1) with coefficient shrinkage (L2), allowing correlated features to be retained while eliminating redundant ones that contribute little to prediction performance. Tree-based: includes decision trees, random forests, and gradient boosting machines, where features are ranked based on their contribution to model decision splits (eg, Gini importance or SHAP values). Features with minimal impact can be removed using a predefined importance threshold.
Model design and augmentation:	
Modelling clinical outcomes	The most stable and informative radiomic and clinical features should be selected to build models.^62^ While there is no consensus on the optimal approach, the RQS offers some guidance.^20^ Different techniques should be tested to identify the model that best captures the underlying patterns in the dataset. Comparisons should consider performance metrics, interpretability, and complexity. In addition, factors such as local experience, previously published research, and the specific outcome of interest can influence the choice of modelling techniques. Therefore, the rationale for model selection should be clearly documented. Documentation should include the choice of statistical software used, its version, and key parameter settings. Time-to-event models have traditionally been built using cox regression-based methods.^68^ LASSO regression analysis can be used to improve accuracy and performance.^68^ Supervised and unsupervised ML methods are able to analyse large amount of data within a multidimensional variable space and as a result demonstrate improved performance.^70^^,^^71^ However, there is no agreed recommendation as to which ML method should be used.^72^ Supervised methods are at higher risk of producing overfitted models that lack accuracy and perform poorly during validation steps.^72^ Unsupervised methods are less likely to produce overfitted models. However, they are computationally more complex, can detect meaningless patterns in the data, have a longer processing time, and there can be uncertainty about how data was clustered.^73^
Hyperparameter configuration/tuning or optimization	Hyperparameters are adjustable components of a model that include the structure, the number of categorization clusters, and the learning rate.^74^ Model performance can be refined and improved by adjusting these parameters using optimization algorithms.^75^ To prevent overfitting of the training data, the generalizability of the hyperparameters can be assessed using a separate test set or nested cross-validation.^72^
Multi-modality analysis, timepoints, delta-radiomics and dosiomics	Previous studies have demonstrated superior model performance when built using features extracted from different imaging modalities and from different timepoints.^75^^,^^76^ Delta-radiomic studies can track changes in feature values between scans. Dosiomics is similar to radiomics but instead involves analysing the radiotherapy dose distribution data and spatial dose features are extracted.^77^ Studies that combine radiomic and dosiomic features have demonstrated improved performance.^78^
Discrimination statistics	Discrimination is a statistical term that describes the functioning of a model and how well it predicts the chosen outcome.^79^ For example, if a model predicts tumour control, then good discrimination is demonstrated by being able to correctly identify those patients whose tumour will be controlled. Performance is summarized by combining the true positive rate (sensitivity) with the false positive rate (1-specificity). Methods to summarize this information include graphically using ROC curves or numerically by calculating the C-statistic or area under the curve.
Calibration statistics	Calibration is a statistical term that describes the ability for a model to produce the expected outcomes based on the true underlying probability of the data.^80^ For example, if a model predicts tumour control rate as 85%, then the tumour control rate should actually be 85%. The quality of the calibration can be assessed by plotting predicted probabilities against those observed and calculating different intercepts and slopes. It can also be assessed with “calibration-in-the-large” measures otherwise known as mean calibration.
Internal validation:	
Cross-validation resampling	Cross-validation is a form of resampling used for internal validation that can assess a model’s accuracy and generalization. The training data (approximately 80% of the available data) are used to build the model.^79^ The test dataset (the rest of the available data) is then used to validate it. This process can be repeated multiple times to produce multiple models and to assess consistency and to produce a more representative model. Exhaustive or non-exhaustive methods are used: Exhaustive methods are more complete as all combinations and iterations of a dataset are assessed. They are time and resource intensive unless automated ML processes are used. Non-exhaustive methods are less thorough but easier to perform as they only use a finite number of combinations and iterations of a dataset.
Bootstrap resampling	Bootstrapping is another form of resampling that can be used as a method of internal validation.^81^ It describes a process where a random number of patients within the training dataset are replaced forming a new cohort. This new cohort is then used to assess uncertainty of the model parameters and model performance. Jackknifing or leave-one-out validation is similar, but instead one patient is removed from the training set and model performance is reassessed.^82^ Bootstrapping and jackknifing can be repeated multiple times to assess model performance and similar to cross-validation can be exhaustive or non-exhaustive.

Abbreviations: IBSI = Image Biomarker Standardisation Initiative; ML = machine learning; SHAP = SHapley Additive exPlanations; RQS = radiomic quality score.

## Study analysis and model deployment (RQS 7,12-16)

Model deployment allows performance to be evaluated across different settings using unseen cohorts. It also allows researchers to highlight significant results, discuss the broader impact of their findings, and propose future directions.

### External validation of results

Uses an independent cohort of patients to quantify systematic error.^80^^,^^83^ Confusion matrices and evaluation metrics can be calculated to assess whether a model is truly valid and generalizable. Evaluation metrics are composite measures based on true/false positive/negative rates and include accuracy, recall, and precision. Multiple independent cohorts are preferable and can be prospective, retrospective, real-world registry, or from other studies. Despite being an integral step in model development, it is rarely performed. This is due to the challenges of accessing appropriate cohorts. Models that do not demonstrate validity should still be reported to inform future research.

The importance of this was highlighted by a Maastricht-based research group during their retrospective study on CBCTs.^84^ Predictive models for clinical outcome were built using patients with stage I-IV NSCLC with three independent datasets used for validation. Disappointingly, they were unable to validate their models. They emphasized heterogeneous sampling, variation in image acquisition, and segmentation techniques as reasons for this. Despite the negative results, this paper can be considered an exemplar on the methodology of how to perform longitudinal CBCT radiomic analysis.

### Translation of basic radiomic science

Traditionally, biological biomarker research is driven by preclinical experiments that enables mechanism of actions to be described prior to translation and verification. The notion that a group of voxels can describe underlying microscopic information is not without its critics. Therefore, efforts should be made to provide a biological context of radiomic biomarkers to underlying cellular processes, instead of relying on a black box-like approach to analysis.^85^ This allows researchers to: (1) strengthen the identified association between radiomic biomarker and outcome, (2) gain wider acceptance in the research and clinical communities, and (3) bring radiomic research more in line with the reporting and evaluation standards adopted by other biomarker disciplines.^86^

### Clinical context, utility, and comparison to the gold standard

The results should be discussed considering the study objectives, as well as the current disease and treatment landscape and whether a comparison to the gold standard is being made. This provides important context and prompts a discussion around clinical utility and statistical power. It is important to not overstate non-powered results even when significance is identified. Additionally, it presents an opportunity to highlight the research’s strengths and weaknesses. Patient-reported outcome measures (PROMs) can be used to enhance discussions around patient experience and symptoms. A patient and public involvement (PPI) perspective can be invaluable when discussing impact to ensure relevance. Ultimately, the discussion needs to give a clear steer for future research that remains patient focused.

### Cost-effectiveness analysis

As radiomic analysis uses standard of care imaging and can be highly automated, it has the potential to be highly cost-effective and associated with negligible patient inconvenience. When discussing clinical application, it is essential to consider the financial, time, and resource costs/savings. Any clear benefits should be highlighted to underscore the practical implications of the research. Contemporary healthcare costs have increased dramatically, making it more challenging to fund expensive new interventions. Healthcare funders rely on complex cost-effective calculations that include additional dimensions such as quality of life, which are collected through PROMs. If these data are collected, then experts knowledgeable in health economics can determine the best approach for presenting data. They can also contribute to discussions around the complexities of healthcare costs and potential impact of a radiomic model.

### Publication, open-science, and information sharing

Open-science and -data are key concepts to ensure research is readily accessible to the lung cancer community with minimal barriers and without copyright restrictions. It can include all aspects of the research process, including protocols, methodology, raw data, statistical analysis, and software code. Transparency and collaborative working allow other research groups to evaluate and validate the study outputs more openly. In addition, information sharing and open discussions encourage cooperative working and the development of research networks.

## Discussion

This practical guide, aimed at early career researchers, was crafted through a critical lens, considering the limitations of existing research. It thoroughly examines the current landscape while also delving into future directions. It complements the existing guidelines with a more comprehensive workflow designed to troubleshoot common issues and improve quality. Additionally, it introduces a holistic approach with a focus on patient and stakeholder engagement, setting relevant clinical questions and adopting a more thoughtful practical approach to research output.

Despite numerous published studies, a widely accepted radiomic-based biomarker to guide lung cancer management has yet to emerge.^5^ Limitations of previous studies have been discussed, and in addition, there is often limited discussion of tangible clinical indications. A search on clinicaltrials.gov using the search criteria “lung cancer” and “radiomic” identified 15 registered studies (summary in Supplementary Table 4). Eight of these studies include prospective cohorts,^57^^,^^87–93^ while only four are recruiting patients from more than one institute.^59^^,^^88^^,^^92^^,^^94^ No radiomic-driven randomized studies recruiting patients with lung cancer were identified. One interventional study recruiting patients with sarcoma was identified when using the search criteria “cancer” and “radiomic” and filtering for *interventional study*. The HEAT trial is using an MRI-based hypoxia radiomic model to offer patients diagnosed with sarcoma adaptive radiotherapy.^95^ The slow progress in translating exploratory radiomic studies into interventional studies demonstrates the lack of confidence in the current published research and highlights the importance of having a more standardized approach.

Specific to lung cancer, there are unique challenges related to movement caused by breathing motion. Currently, there are few studies analysing feature stability on CT images acquired during the breathing cycle and comparing stability between diagnostic thorax CT and CBCT performed during radiotherapy. Therefore, additional research is necessary to enhance our understanding of the temporal and spatial stability and reproducibility of radiomic features.

We encourage early career researchers to embrace the approach described in this practical guide when planning and carrying out research. We support ongoing discussion and refinement of methods, which should be promoted through collaborative efforts, a culture of information sharing and adherence to best practices. Additionally, we anticipate greater acceptance and reliance on automated processes to enhance efficiency. It is through the production of high-quality, transparent research that confidence in radiomic features identified can be established, ultimately advancing their potential role in realizing the goals of personalized medicine.

## Supplementary Material

tqaf051_Supplementary_Data
