# Supplementary material for: Mastering CT-based radiomic research in lung cancer: a practical guide from study design to critical appraisal
Source: Br J Radiol. 2025 Mar 18;98(1169):653–68. doi: 10.1093/bjr/tqaf051 (PMC12012345; doi:10.1093/bjr/tqaf051)
Supplement: tqaf051_Supplementary_Data [file tqaf051_supplementary_data.zip › tqaf051_Supplementary_Data/Appendix table 1.docx]

| **Clinical Outcome** | **Patient-related features** | **Tumour-related features** | **Treatment-related features** |
| --- | --- | --- | --- |
| *Local control rates* | Age  Gender  Performance status  Symptoms  BMI & weight loss  Smoking status  Neutrophil/lymphocyte ratio | Stage  Histology  Tumour volume  Tumour site  Genomic markers (e.g PD-L1 and driver mutation status)  SUVmax on PET-CT scan  Texture on CT scan | Dose fractionation  Radiotherapy technique  Chemotherapy  Consolidation immunotherapy  Extent of resection |
| *Distant control rates* | Age  Gender  Performance status  BMI & weight loss  Comorbidity  Platelet/lymphocyte ratio  Albumin  C-reactive protein | Stage  Histology  Tumour volume  Tumour site  Genomic markers (e.g PD-L1 and driver mutation status)  SUVmax on PET-CT scan  Vascular invasion | Dose fractionation  Radiotherapy technique  Chemotherapy  Consolidation immunotherapy  Extent of resection |
| *Toxicity rates* | Age  Gender  Performance status  Race  BMI & weight loss  Lung function/volume  Genomic features | Stage  Histology  Tumour volume  Tumour site in relation to organ at risk | Dose fractionation  Radiotherapy technique  Dose to organs at risk  Chemotherapy  Consolidation immunotherapy  Surgical procedure |

**Appendix table 1.** *Critical patient-, tumour- and treatment-related features associated with clinical outcomes following curative-intent treatment for lung cancer* (7,8)*.*
