# Supplementary material for: Mastering CT-based radiomic research in lung cancer: a practical guide from study design to critical appraisal
Source: Br J Radiol. 2025 Mar 18;98(1169):653–68. doi: 10.1093/bjr/tqaf051 (PMC12012345; doi:10.1093/bjr/tqaf051)
Supplement: tqaf051_Supplementary_Data [file tqaf051_supplementary_data.zip › tqaf051_Supplementary_Data/Appendix table 2.docx]

| **No:** | **Checkpoint** | **Description** | **Scoring system** | **Score** |
| --- | --- | --- | --- | --- |
| **1.** | Image protocol quality | Documenting image protocol used or using public image protocols | Protocols well documented  Public protocol used  None | +1  Or  +2 |
| **2.** | Multiple segmentation methods | Includes different physicians, algorithms, software, perturbation with random noise, segmentation at different breathing cycles. | Yes  No | +1 |
| **3.** | Phantom study on all scanners | Deter inter-scanner differences and vendor-dependent features | Yes  No | +1 |
| **4.** | Imaging at multiple time points | Using images at different time points to assess temporal variability in images. | Yes  No | +1 |
| **5.** | Feature reduction or adjustment for multiple testing | Reduces risk of overfitting. | Either measure is implemented  Neither measure is implemented | -3  Or  +3 |
| **6.** | Multivariable analysis with non-radiomic features. | Generates a more holistic model and correlating between radiomic and non-radiomic features | Yes  No | +1 |
| **7.** | Detect and discuss biological correlates | Demonstration of phenotypic differences to improve understanding of radiomics and biology | Yes  No | +1 |
| **8.** | Cut-off analysis | Using median, previously published cut-off or report a continuous risk variable. | Yes  No | +1 |
| **9.** | Discrimination statistics and resampling techniques. | Discrimination statistics such as C-statistic, ROC curve and AUC alongside p-values and confidence intervals.  Resampling techniques such as bootstrapping or cross-validation | Discrimination statistic reported.  And resampling technique.  None | +1  Or  +2 |
| **10.** | Calibration statistics | Calibration statistics such as in-the-large/slope or calibration plots, alongside p-values and confidence intervals.  Resampling methods can also be used. | Calibration statistic reported.  And resampling technique.  None | +1  Or  +2 |
| **11.** | Prospective study | Registered in a trial database. | Yes  No | +7 |
| **12.** | Validation | Validation using datasets from different institutes or validating a previously published signature | No validation.  Validation from same institute  Validation from another institute  Validation from two distinct institutes  Validates a previously published signature.  Validation from three distinct institutes. | -5  To  +5 |
| **13.** | Comparison to gold standard | Compares radiomic based model to current standard, e.g. TNM, to demonstrated value of radiomics | Yes  No | +2 |
| **14.** | Potential clinical utility | Report on current and/or potential application of the model in a clinical setting | Yes  No | +2 |
| **15.** | Cost-effectiveness analysis | Report on the cost-effectiveness of the clinical application | Yes  No | +1 |
| **16.** | Open science and data | Make code and data publicly available | Scans  Region of interest segmentation  Code  Radiomic features | +1 to +4 |

**Appendix table 2.** *The RQS version 1.0 16-point framework* (20)*.*
