# Supplementary material for: Mastering CT-based radiomic research in lung cancer: a practical guide from study design to critical appraisal
Source: Br J Radiol. 2025 Mar 18;98(1169):653–68. doi: 10.1093/bjr/tqaf051 (PMC12012345; doi:10.1093/bjr/tqaf051)
Supplement: tqaf051_Supplementary_Data [file tqaf051_supplementary_data.zip › tqaf051_Supplementary_Data/Appendix table 3.docx]

| **Morphological/shape features** | |
| --- | --- |
| Shape-based features | Describes features, such as compactness and sphericity, that measure the difference between a standardised shape and ROI volume.  Due to similarities between these features, they are often highly correlated and therefore a source of feature redundancy. |
| Length-based features | Includes features that are calculated using different ROI diameters or axes and distances between intensity regions. |
| Composite measures | Are summary ratios based on combining two morphological features. This includes the surface area to volume ratio or elongation which is the ratio between major and minor principal axis length.  Other composite measures are based on volume and area densities or measures that combine volume and intensities. |
| **First order/intensity features** | |
| Local intensity features | These features are calculated by analysing the voxel intensity within a predefined 3D volume around a central voxel. Voxels that originate from outside the ROI can be included. Statistical analysis includes measures of distribution, such as mean, median, range, deviation, dispersion and kurtosis. |
| Intensity histogram features | Describes features that are calculated from discretised intensity summary histograms and statistics. Summary statistics like *local intensity features* can be produced alongside specific histogram measures such as uniformity and gradient measures. |
| Composite intensity-volume histogram features | Describes features that combine a volume and intensity feature. Summary measures such as volume at intensity, intensity at volume fractions and differences between fractions can be calculated. |
| **Second (or higher) order/textural features** | |
| Grey level co-occurrence matrix (GLCM) | GLCM assesses co-occurrence or combinations of grey levels of voxels along a direction vector within a volume. Vector distance weighting can be performed although the IBSI recommends against this due to reproducibility issues. Summary statistics based on joint, sum, clustering, correlation, moments and difference can be calculated. |
| Grey level run length-based features (GLRLM) | GLRLM features measures lengths of consecutive voxels with the same grey level along a vector. Distance weighting is also not supported by the IBSI. Summary features focusing on specific lengths of runs or grey level ranges can be calculated. Composite measures combining length and grey level data can also be produced. |
| Grey level size zone-based features (GLSZM) | GLSZM features are a measure of zones of voxels with the same grey level intensity. Like GLCM and GLRLM, summary and composite features that focus on specific grey levels and different sized zones can be generated. |
| Grey level distance zone-based features (GLDZM) | GLDZM features combine GLSZM with position. Grey level zones with the same intensity are grouped together if they are the same distance from the ROI mask. Summary features based on different distances and intensities can be produced. |
| *Neighbourhood grey tone difference-based features (NGTDM)* | NGTDM features summarise information describing differences in grey level intensity with reference to neighbouring voxel intensities. This includes measures that describe strength and complexity. |
| *Neighbouring grey level dependence-based matrix (NGLDM)* | NGLDM features describe the coarseness of the overall texture of voxels within the ROI and has similar structure to GLRLM, GLSZM and GLDZM features. |

**Appendix table 3.** *Table summarising key radiomic features by type.*
