# Supplementary material for: Mastering CT-based radiomic research in lung cancer: a practical guide from study design to critical appraisal
Source: Br J Radiol. 2025 Mar 18;98(1169):653–68. doi: 10.1093/bjr/tqaf051 (PMC12012345; doi:10.1093/bjr/tqaf051)
Supplement: tqaf051_Supplementary_Data [file tqaf051_supplementary_data.zip › tqaf051_Supplementary_Data/Appendix table 4.docx]

| **Study detail** | **Patients** | **Primary end point** | **Other relevant detail** | **Comments** |
| --- | --- | --- | --- | --- |
| **NCT06331975** (86)  *Prospective study*  Milan, Italy | N=91 (actual as of 26/03/2024).  Eligibility: NSCLC, surgically managed, suitable for driver mutation assessment. | To assess for associations between EGFR, ALK, KRAS status and clinical outcome. | To build a radiomic model to predict driver mutation status and prognosis. | Liquid biopsies mentioned.  No stage restriction within eligibility criteria. |
| **TOP-RLC study** (87)  **NCT04452058**  *Prospective study*  Guangzhou, China  Zhoushan, China | N=500 (estimated upon completion)  Cohort I eligibility: stage I adenocarcinoma, surgically managed.  Cohort II eligibility: advanced NSCLC, treated with immunotherapy. | Cohort I: To build a radiomic model to distinguish between precancerous lesions and adenocarcinoma. | Cohort II: To build a radiomic model to predict response to palliative immunotherapy. | Peri-tumour (5mm) features included.  Please note: two distinct patient cohorts being assessed in parallel. |
| **NCT06405815** (88)  *Prospective study*  Nanjing, China | N=200 (actual as of 09/05/2024).  Eligibility: stage III NSCLC, surgically managed. | To build a radiomic model to predict high-risk surgical margins and prognosis. | High risk surgical margins defined as R1 or R2 resections. | Peri-tumour (3mm) features included. |
| **NCT03940846** (95)  *Retrospective study*  Maastricht, Netherlands | N=650 (estimated upon completion).  Eligibility: NSCLC who underwent biopsy. | To build a CT-based radiomic model to identify adenocarcinoma histology. |  | External validation highlighted (3 independent cohorts (n=1053)).  No stage or treatment restriction within eligibility criteria. |
| **AI-SONAR study** (96)  *Retrospective study*  London, United Kingdom | N=1000 (estimated upon completion).  Eligibility: previous radically treated malignancy, new lung nodule. No specific treatment modality. | To build a radiomic model to predict whether new lung nodules are benign or malignant. | Nodules will be defined as benign, metastatic or new primary lung cancer. |  |
| **NCT01585545** (89)  *Prospective study*  Seoul, South Korea | N=500 (estimated upon completion).  Eligibility: NSCLC. Treated with surgery or drug-based therapies. | To identify prognostic radiomic or genomic markers predictive of clinical outcome. | To identify relationships between radiomic and genomic markers.  To identify radiomic markers that predict post-surgical lung function. | No stage or treatment-intent restriction within eligibility criteria. |
| **NCT03648151** (93)  *Retrospective study*  Anhui, China  Shanxi, China | N=1000 (actual as of 23/07/2020).  Eligibility: lung cancer, underwent PET-CT scan. | To build a radiomic model that predicts overall survival. | Radiomic features include those relating to SUV on PET-CT scan. | External validation highlighted.  No stage or treatment restriction within eligibility criteria. |
| **Blue Sky Radiomics study** (59)  **NCT4364776**  *Prospective and retrospective study*  Pavia, Italy | N=100 (actual as of 28/09/2023)  Eligibility: stage III NSCLC. Treated with concurrent chemoradiotherapy followed by consolidation durvalumab. | To compare performance of two existing radiomic models that predict progression free survival. | Delta-radiomic component: CT scan pre and post chemoradiotherapy included. | Preliminary results identified 6 radiomic features predictive of progression free survival (97). Opportunity to improve performance of existing models through integration of newly identified features. |
| **NCT03954847** (98)  *Retrospective study*  Wuhan, China | N=1000 (estimated upon completion)  Eligibility: NSCLC. | To identify radiomic features that predict overall survival. | To identify radiomic features that predict progression free survival. | No stage or treatment restriction within eligibility criteria. |
| **SMAC-2 study** (90)  **NCT04315753**  *Prospective study*  Milan, Italy | N=2000 (estimated upon completion).  Eligibility: Age ≥55 years old, significant smoking history. No symptoms of or previous lung cancer diagnosis. Patients will then enter lung cancer screening program with aim to recruit 50 patients with screen detected lung cancer, the rest of the cohort to be used as a control. | To assess the role of molecular, liquid biopsy and radiomic model to support early detection of lung cancer through lung cancer screening. | Comparative cancer cohort, not identified through screening (n=70, stage I-II lung cancer being managed surgically).  Baseline and at 4 months liquid biopsy.  Comparative non-cancer cohort, recruited through screening who do not develop lung cancer. | Liquid biopsy analysis includes circulating tumour cells, cell-free tumour DNA and tumour exosomes. |
| **PEMBRONIC study** (99)  **NCT05996263**  *Retrospective study*  Brest, France | N=75 (estimated upon completion).  Eligibility: stage IV NSCLC, tumour PD-L1 ≥50%. Treated with pembrolizumab monotherapy. | To build a model that combines KEAP1/NFE2L2 mutations and radiomic features in predicting progression free survival. | To build a model that combines KEAP1/NFE2L2 mutations and radiomic features in predicting overall survival. | Mention of robustness check of predictive model using 2 blind reviewers. |
| **PANU study** (61)  **NCT04276025**  *Retrospective study*  Bayreuth, Germany  Hof, Germany  Beijing, China | N=2000 (actual as of 19/02/2020).  Eligibility: surgically treatable NSCLC. Mutation testing, circulating tumour cell and PET-CT available. | To identify robust and radiomic features using two different published methods. | To evaluate the relationship between mutation testing, circulating tumour cells and PET-CT features with clinical outcome. | Use of international independent cohort to validate findings. |
| **I3LUNG study** (91)  **NCT055537922**  *Prospective and retrospective study*  Chicago, USA  Athens, Greece,  Gerusalemme, Israel  Barcelona, Spain | Retrospective cohort N=2000.  Prospective cohort N=200 (estimated upon completion).  Eligibility: stage IIIB/C-IV NSCLC, previously treated with immunotherapy. | To use the retrospective cohort to build a model that predicts response to immunotherapy using clinical and radiomic features. | To use the retrospective cohort to build a model that predicts progression free survival/overall survival using clinical and radiomic features. | Use of prospective international cohort to validate findings.  Explicit mentioning of health economic, quality of life measures and how the information could be used to build a clinically useful decision support tool. |
| **VIGILANCE study** (92)  **NCT06086574**  *Prospective study*  Manchester, United Kingdom | N=80 (estimated at time of completion).  Eligibility: stage III NSCLC, being treated radically with radiotherapy. | To build a prognostic model built using circulating tumour DNA, radiomic features and PROM data to predict overall survival, tumour control and early tumour relapse. | To describe longitudinal changes in circulating tumour DNA, radiomic features and PROM data before, during and for 1 year following completion of radiotherapy.  To assess for associations between features as described. | Longitudinal aspect of data collection before, during and after radiotherapy. |
| **ROSALIND study** (100)  **NCT06160596**  *Retrospective study*  Villejuif, France | N=1020 (estimated at time of completion).  Eligibility:  Patients with extensive stage small cell lung cancer, metastatic pancreatic adenocarcinoma or glioblastoma IDHwt split into two groups: long term and normal survival. | Small cell lung cancer: to build a model that predicts long term survival using histological, radiomic, genomic, transcriptomic, proteomic and clinical features. | Similar objectives with pancreatic cancer and glioblastoma cohorts. | Long term survival in small cell lung cancer defined as ≥5 years from diagnosis. |

**Appendix table 4.** *Summary of current radiomic lung cancer studies registered on the Clinical Trials database* (36)*.*
